# Extracellular vesicle production: A bidirectional effect in the interplay between host and *Candida* fungi

**DOI:** 10.1016/j.crmicr.2024.100255

**Published:** 2024-06-22

**Authors:** Kamila Kulig, Maria Rapala-Kozik, Justyna Karkowska-Kuleta

**Affiliations:** Department of Comparative Biochemistry and Bioanalytics, Faculty of Biochemistry, Biophysics and Biotechnology, Jagiellonian University, Gronostajowa 7, 30-387 Kraków, Poland

**Keywords:** Extracellular vesicles, Fungi, *Candida*, Inflammation, Host response, Macrophages, Epithelium

## Abstract

*Candida* fungi exploit various virulence strategies to invade the human host, while host cells employ diverse mechanisms to maintain homeostasis and respond to infection. Extracellular vesicles (EVs) are integral components of the multifaceted landscape of host-pathogen interactions, with their abundant production by all contributors involved in these complex and dynamic relations. Herein, we present the current state of knowledge regarding the host response by releasing EVs in reaction to *Candida*, as well as the influence of fungal EVs on the functionality of the confronted host cells. Fungal vesicles contribute to enhanced adhesion of pathogens to human cells as evidenced for *C. auris*, and may modulate the production of several cytokines, including IL-6, IL-8, IL-10, IL-12p40, TGF-β and TNF-α, thereby exerting pro-infective and pro-inflammatory effects, as described for *C. albicans* and other *Candida* species. Whereas the biosynthesis of EVs by host cells can dynamically modulate the proliferation and viability of fungal cells and affect the candidacidal functionality of other effector cells. The reciprocal influence of EVs from host cells and *Candida* pathogens is a key focus, explaining their significant role in cell signaling and interkingdom communication.

## Highlights

• *Candida* pathogenic fungi use various strategies to invade human host, including production of extracellular vesicles (EVs).
• Host cells respond to *Candida* infection with diverse mechanisms, that modulate conditions at the infection site.
• EVs play a crucial role in the complex interactions between the host and the pathogen.
• EVs from pathogens and from host cells influence cell signaling and interkingdom communication.

## Graphical abstract

**Figure:**
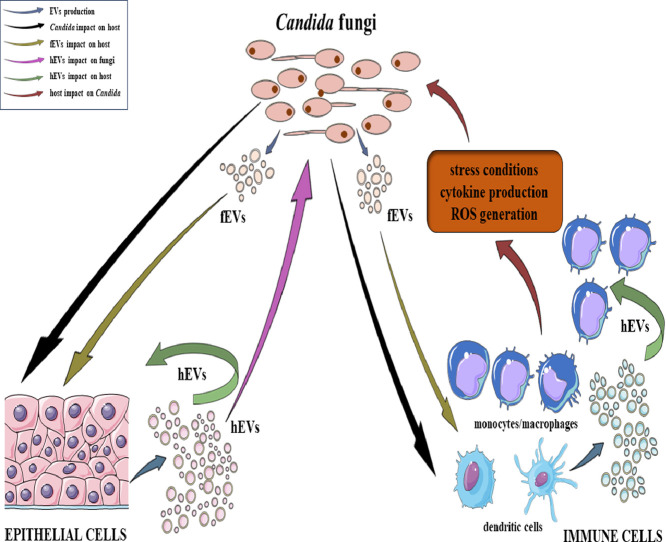

## 1 Introduction

Human cells exploit numerous adaptive mechanisms, that are responsible for the maintenance of homeostasis, dynamic response to different environmental stimuli, and effective intercellular communication upon the onset of varied pathological conditions (Meizlish et al., 2021). In reaction to infection triggered by microbial pathogen, the host inflammatory response is actively developed, with further modulation of the conditions in the infectious milieu (Chaplin, 2010). Different types of human cells facing the invader dynamically react to infection by activating various, often interdependent processes. Epithelial cells, which are often the first to respond due to their localization, release cytokines, chemokines, and a variety of signaling molecules, generate reactive oxygen species (ROS) and modify intercellular adhesion properties by altering the profile of intercellular junctions ‒ tight junctions, gap junctions, and desmosomes (Schleimer et al., 2007). After epithelium stimulation, several types of immune cells are activated and recruited to the infection site, with subsequent phagocytosis, oxidative burst and ROS generation, release of proinflammatory cytokines and chemokines, production of various antimicrobial proteins and peptides, and formation of extracellular traps (Naglik, 2014; Netea et al., 2015). This comprehensive immune reaction creates a complex microenvironment that should favor removal of the pathogens’ cells and effective clearance of the infection. However, as pathogenic microorganisms have evolved numerous adaptive mechanisms to evade host defense system, the final effect depends on the successful manipulation of the balance by both sides. A recently discovered mechanism involved in these mutual interactions is the production of extracellular vesicles (EVs) ‒ cell-derived, membrane-surrounded vesicles that transport genetic material, proteins, peptides, lipids, polysaccharides, and small metabolites ‒ which are produced by all known cell types (Raposo and Stoorvogel, 2013; Zaborowski et al., 2015). EVs are involved in intercellular communication not only within the same species, but also between cells representing different kingdoms of life (Woith et al., 2019; Bitencourt et al., 2022a). They may be important connectors that play a key role in the pathogenesis of infection (Wang et al., 2018; Zhang et al., 2018). Importantly, the composition of EVs is crucial for their functional properties and depends on external stimuli and environmental conditions that affect the state of the cell (de Toledo Martins et al., 2018; Jin et al., 2022; Liu and Wang, 2023).

EVs produced by fungi pathogenic for humans were first isolated and characterized in 2007 by Rodrigues et al. (Rodrigues et al., 2007) for *Cryptococcus neoformans*, and then, the essential involvement of EVs in the physiology and virulence of fungal cells of different species has been repeatedly described (as summarized in Joffe et al., 2016; Herkert et al., 2019; Garcia-Ceron et al., 2021). The production of EVs has also been demonstrated for several species of pathogenic yeast-like fungi of the genus *Candida* (Rodrigues et al., 2008; Gil-Bona et al., 2015; Vargas et al., 2015; Karkowska-Kuleta et al., 2020a; Zamith-Miranda et al., 2021). *Candida* yeasts are responsible for widespread, bothersome, and recurrent superficial infections characterized by a high morbidity and localized in different host niches, including the oral cavity, gastrointestinal tract or lower genital tract, but they are also the cause of serious systemic diseases with a mortality rate in the range of 40–55 % (Millsop and Fazel, 2016; Bongomin et al., 2017; Pappas et al., 2018; Yano et al., 2019). *Candida albicans* is the species responsible for approximately 50 % of candidal diseases worldwide; however, the incidence of diagnosed infections caused by other emerging pathogens of this genus ‒ non-*albicans Candida* species ‒ is constantly increasing and alarming due to their different pathogenicity mechanisms and resistance to antifungal drugs (Lee et al., 2021). The group of the most frequently identified non-*albicans Candida* species potentially pathogenic to humans includes *C. parapsilosis, C. tropicalis, C. dubliniensis,* and *C. auris* (Lamoth et al., 2018; Pfaller et al., 2019; Gómez-Gaviria et al., 2022). Previously, the genus *Candida* also included *C. glabrata,* which is currently known as *Nakaseomyces glabratus*, and is still considered a species that poses a significant threat to human health (Kidd et al., 2023).

The development of both, superficial and deep-seated candidal infections is associated with the use of a number of virulence factors and mechanisms that allow fungi to effectively adhere to host tissues followed by their destruction and invasion (Mayer et al., 2013). They are also involved in evading and combating the host immune response. The mechanisms related to the pathogenicity of *Candida* fungi are based on the morphological transition between yeast-like cells and filamentous forms (hyphae and pseudohyphae), enhanced stress resistance, secretion of hydrolytic enzymes and toxins, surface display of adhesive proteins, biofilm formation, quorum sensing and production of EVs (Karkowska-Kuleta et al., 2009; Staniszewska, 2020; dos Santos and Ishida, 2023). *Candida* vesicles are carriers of many important virulence factors including secreted aspartic proteinases, adhesins from Als family, or moonlighting proteins also involved in the pathogenesis of fungal infection (Gil-Bona et al., 2015; Vargas et al., 2015; Konečná et al., 2019; Zarnowski et al., 2021). Fungal vesicles also carry RNA molecules as their cargo, which may affect the gene expression in the host cells and other, co-localized fungal cells (Peres da Silva et al., 2015; Zamith-Miranda et al., 2021; Bitencourt et al., 2022b).

The compositional attributes and structural properties of EVs released by both, the host, and the pathogen, are influenced by a multitude of factors, including environmental conditions, the state of the cells, stage of infection, and the influence of chemical and biological factors in the surroundings. Characterizing these structures is therefore currently under intense investigation (see Table 1). Once initiated, the interplay between host cells and pathogens triggers further dynamic changes in the infectious site that modulate the communication between the opposing cells. Therefore, the main objective of this review will be to focus on the mutual impact of EVs released by host cells (hEVs) on fungal cells and EVs from fungi (fEVs) on diverse types of host cells, in particular to highlight the challenges and issues that should be further explored in depth to better understand these complex relationships and to facilitate the development of strategies for their regulation. The novel exploration of the reciprocal effects of host and fungal EVs presented herein is also focused on the escalation of cellular responses during inflammation, driven by the bidirectional communication via EVs that continually adapt to inflammatory stimuli.

**Table 1:** The range of characteristics of host-derived EVs (hEVs) and fungal-derived EVs (fEVs) mentioned in this review.

**Source of EVs**	**Range of EVs characteristics**	**Reference**
**hEVs**		
ARPE-19 epithelial cells infected with *C. albicans*L-614/2017	Size and concentration (by DLS) morphology (by SEM) proteomic composition surface composition	Gandhi and Joseph, 2022
Mice eyeball cells infected with *C. albicans*L-614/2017	Size (by DLS) proteomic composition cytokine composition and concentration	Gandhi et al., 2022
THP-1 macrophage-like cells infected with *C. albicans* SC5314	Morphology (by TEM) size (by DLS) proteomic composition and concentration	Reales-Calderón et al., 2017
Blood-derived monocytes infected with *C. albicans* SC5314	Concentration (by DLS) proteomic composition cytokine composition and concentration	Halder et al., 2020
	miRNA composition	Halder et al., 2022
**fEVs**		
*C. albicans* ATCC 64,548 grown in the presence of menadione	Lipid composition	Trentin et al., 2023
*N. glabratus, C. parapsilosis* and *C. tropicalis* hyphal cells	Surface composition	Karkowska-Kuleta et al., 2020a
*C. albicans* ATCC 90,028 and clinical strain cultured in nutrient limited conditions	Proteomic composition	Konečná et al., 2019
*C. auris* MMC1, B8441 and B11244 cultured in the presence of caspofungin	Size and concentration (by TEM and NTA analysis) proteomic composition	Amatuzzi et al., 2022
	mRNA sequence identification	Munhoz da Rocha et al., 2021

## 2 EVs impact on infection caused by *Candida* fungi

There are at least four major consecutive stages during the infection caused by *Candida* fungi – adhesion, invasion, tissue damage, and further dissemination (Wang et al., 2012). In the course of these processes, different molecules produced by the host cells, including proteins or peptides, have a substantial impact on the fungal cells and their virulence mechanisms. The potential influence of various plasma proteins, such as plasminogen, fibronectin, kininogen, vitronectin or complement factors on the adhesive properties of different *Candida* species has been frequently proposed (Karkowska-Kuleta et al., 2010, 2011, 2016, 2017, 2020b; Kozik et al., 2015; Seweryn et al., 2015; van der Wielen et al., 2016; Zajac et al., 2016; Satala et al., 2020, 2023). Furthermore, also the contribution of different host proteins to the formation of biofilms by *C. albicans* cells has been demonstrated (Nett et al., 2015)*.* However, not only soluble individual molecules may be crucial in such interactions, but also those transported, packaged, and assembled in EVs may play an important role (Sabatke et al., 2023).

Generally, the first stage of fungal infection is considered to be the contact of microbial cells with epithelial cells initiating the adhesion process, and the EVs produced by the latter (host EVs, hEVs) may be significantly involved in affecting fungal cells. As demonstrated by Zhao et al. (2022), hEVs released by the human oral mucosal epithelial cell line Leuk-1 were reported to have an effect on *C. albicans* cells by inhibiting their growth and hyphae formation after 12 h, and microcolony formation after 24 h and 48 h of contact, in a concentration-dependent manner (Zhao et al., 2022). The important participation and involvement of hEVs released by Leuk-1 epithelial cells during *Candida* infection was demonstrated in experiments using Leuk-1 cells in the presence of a specific inhibitor of exosome release ‒ selective inhibitor of neutral sphingomyelinase GW4869 (Zhang et al., 2022; Zhao et al., 2022). The effect of Leuk-1-EVs on *C. albicans* was observed in vitro as morphological changes that led to the damage of yeast cells, as *C. albicans* cells incubated with hEVs were less smooth and regular in shape than untreated hyphal cells. Moreover, in the mouse model of oral candidiasis, the application of Leuk-1-EVs resulted in a noticeable antifungal effect limiting development of *C. albicans* infection (Zhao et al., 2022). These observations confirm the synergistic anti-*Candida* action of epithelial cells and the EVs they produce.

However, the development of the infection caused by *C. albicans* cells could also be stimulated by fungal EVs (fEVs) affecting the host cells of different origin. As demonstrated by Wei et al. (2023), the damage caused by *C. albicans* cells to macrophages from the RAW 264.7 murine cell line, human oral keratinocytes (HOK), human squamous cell carcinoma epithelial cells from the TR146 cell line and gingival epithelial cells (HGEC) during invasion was synergistically enhanced in the presence of fEVs derived from fungi cultured under conditions that promote hyphal formation. The destructive effects observed individually, on host cells only, or on fEVs only, were less pronounced or insignificant. It has been shown that the regulation of *C. albicans* growth by the l-arginine/nitric oxide pathway mediated by produced fEVs, correlates with the observed host cell damage (Wei et al., 2023), indicating the interdependence of cell virulence on the fEVs they produce.

**Fig. 1:**
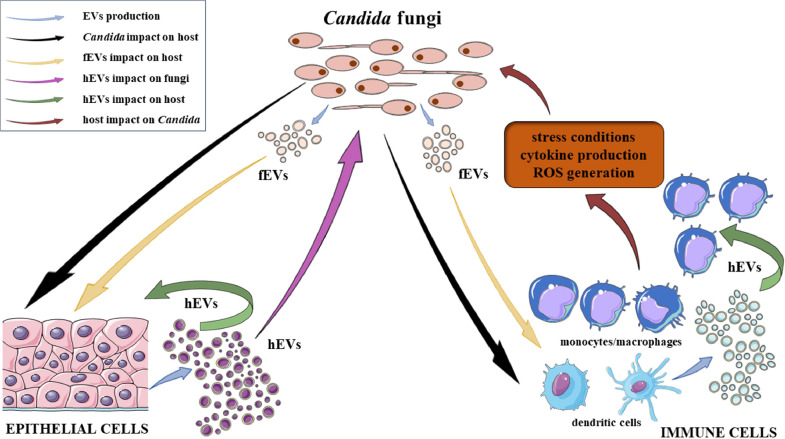
The schematic representation of the interactions and effects of EVs produced by the host (referred to as host EVs, hEVs) and by fungi of the genus *Candida* (referred to as fungal EVs, fEVs) documented to date. These influences are exerted on both fungal cells and host epithelial and immune cells, also taking into consideration the challenge of modulating the interaction milieu subsequent to the process of the initiation of the immune system activation and the further development of inflammation. The figure was created in part using Servier Medical Art, provided by Servier, and licensed under a Creative Commons Attribution 3.0 Unported license.

The above examples highlight the contribution of EVs released by the host on *Candida* cells and by the pathogen on host cells during the infection. However, the underlying mechanisms governing these interactions and their immediate and distant effects are still intricate and remain largely elusive. The mechanisms that have been elucidated to date are discussed in the following sections and reviewed in Fig. 1.

## 3 Effect of *Candida* cells on EVs released by the host

An interesting example of the impact of *Candida* cells on the host response and the release of hEVs is the involvement of fungi as a factor complicating the interactions between primary keratinocytes and dendritic cells in the course of atopic dermatitis (AD) (Kobiela et al., 2022). During the development of superficial candidiasis in patients with AD, which resulted in the exposition of host keratinocytes to *C. albicans* cells and AD-related cytokines, host cells in response to fungal infection released hEVs with changed surface glycosylation pattern, specifically consisting of increased expression of sialic acid forms (Kobiela et al., 2022). The consequence of this phenomenon was the modulation and enhancement of the interactions between keratinocyte-derived EVs and the inhibitory Siglec‒7 and ‒9 receptors present on dendritic cells. This could be one of the possible strategies used by *C. albicans* for immune evasion, due to indirect triggering of the inhibition of the NF-κB-dependent Toll-like receptor 4 (TLR4) signaling pathway (Delaveris et al., 2021; Kobiela et al., 2022).

In the studies presented by Gandhi and Joseph (2022), the production of hEVs differentiated in size, number and proteomic cargo by epithelial cells endangered with fungal invasion has been demonstrated, in comparison to control hEVs produced by healthy cells. After 24 h of infection initiated by *C. albicans* strain L-614/2017, epithelial cells ARPE-19 produced larger hEVs with an average diameter of 126.4 ± 20 nm compared to the 86 ± 12 nm diameter of the exosomes released by healthy cells, as measured by dynamic light scattering (DLS) technique, and the amount of about 4 **×** 10^7^ EVs and 2 **×** 10^7^ EVs, respectively (Gandhi and Joseph, 2022). The analysis of hEVs morphology with scanning electron microscopy (SEM) confirmed the spherical shape and diameters of 782 and 500 nm, respectively, for hEVs derived from infected and uninfected cells (Gandhi and Joseph, 2022). The surface of hEVs produced by infected cells was enriched in the vesicular tetraspanin markers CD9 and CD81 (Gandhi and Joseph, 2022), involved in cell signaling and adhesion and considered essential for the process of sorting and packaging molecules into hEVs and subsequent vesicle uptake (Gandhi and Joseph, 2022; Neda et al., 2023). Both proteins have also been reported to be highly expressed in cancer cells (Neda et al., 2023) and are considered to be involved in preventing senescence and inflammation by maintaining the expression of the SIRT1 gene encoding NAD-dependent deacetylase sirtuin-1; although, the exact mechanisms and implications of this are complex and still under investigation (Jin et al., 2018). One of the functions of the CD81 protein is to participate in the migration of immune cells, but also cancer cells (Tejera et al., 2013), and cell cycle control (Song et al., 2004). Of the 645 proteins identified in the proteomic analysis of hEVs produced by cells infected with *C. albicans*, 419 vesicular proteins were differentially expressed, of which 218 were upregulated, including proteins involved in translation, protein folding, proteasome-mediated ubiquitin-dependent protein degradation, proteasomal protein degradation, activation of the innate immune response, and cellular response to IFN-γ, while 201 proteins were downregulated (Gandhi and Joseph, 2022). Some of the upregulated vesicular proteins, including L-lactate dehydrogenase (LDH), MAPK1 (mitogen-activated protein kinase), HSPB1 (heat shock protein beta-1) or FGF2 (fibroblast growth factor 2) were important in pathways such as the MAPK pathway, the mTOR pathway, and the IL-17 pathway. Furthermore, network-pathway analysis for hEVs produced by cells challenged with *C. albicans* demonstrated enrichment in particular pathways including signal transduction, vesicle-mediated transport, membrane trafficking, immune response, metabolism, and cytokine signaling by the immune system, which supports the production of immune regulators. Thus, this phenomenon appears to be essential for the mechanisms of the pathogenesis of *C. albicans* infection related to the interactions with epithelial cells and release of epithelial-derived hEVs (Gandhi and Joseph, 2022).

The subsequent stages of infection involve the interactions between fungi and immune cells. As demonstrated by Reales-Calderón et al. (2017), hEVs derived from THP-1 cells that differentiated into macrophage-like cells, infected with *C. albicans* SC5314 were different from control hEVs. The presence of pathogen cells induced an increase in the number of vesicles released by THP-1 cells, especially in the population of hEVs with a diameter in the range of 100 nm to 1000 nm, and had an impact on the greater diversity in hEVs populations (Reales-Calderón et al., 2017). Microscopic observation revealed both spherical and non-spherical shapes of the hEVs and the proteomic analysis revealed changes in hEVs cargo. The protein amount in the hEVs released by infected immune cells was almost two times higher compared to hEVs from uninfected cells. Of the 719 identified proteins most were associated with the cell membrane, and bioinformatics analysis highlighted 111 identified proteins being secreted via the classical secretory pathway through the endoplasmic reticulum and Golgi apparatus, while 272 of the identified proteins lacked the signal peptide, whereby they could be classified as secreted via non-classical pathway. Proteins contained in hEVs released by *Candida*-infected THP-1 cells were identified as crucial during immune responses, signal transduction, stress response, organization of cytoskeletal, transport, metabolism, nucleic acid processing or ribosome synthesis. Furthermore, the analysis with Western blot showed increase in the amount of vimentin and decrease in the amount of peroxiredoxin 5 and transferrin receptor in hEVs derived from infected THP-1 cells compared to control hEVs. The protein cargo of hEVs particularly represented molecules involved in binding and catalytic activity, in terms of their molecular function. The analysis of the involvement in biological processes indicated hEVs-proteins responsible for regulation and metabolism, but also interaction with cells and organisms, response to stimuli, and immune system processes. Considering the cellular component defined as typical for the occurrence of identified proteins, the most abundant proteins were from cytoplasm, extracellular environment, and chromosomes (Reales-Calderón et al., 2017).

In the same study the immunomodulatory effect of hEVs obtained from infected cells ‒ differentiated THP-1 cells on other THP-1 derived macrophages was observed after internalization of hEVs, in the increased production of the proinflammatory cytokines TNF-α, IL-8, IL-12. In the case of stimulation of undifferentiated THP-1 monocytes with vesicles, the effect of adding hEVs from infected cells was not as significant as for stimulation of macrophage-like cells, but the immunostimulatory potential was higher than for hEVs derived from uninfected cells and was associated with the production of TNF-α (Reales-Calderón et al., 2017). The observed secretion of cytokines was the effect of the activation of ERK and p38 kinases and the capability to activate kinases was slightly higher for hEVs derived from infected THP-1 cells. An increase of the candidacidal activity of THP-1 macrophages after incubation with hEVs was observed, with a comparable effect for both types of hEVs ‒ from infected and uninfected cells (Reales-Calderón et al., 2017).

Furthermore, as shown by Halder et al. (2020), blood-derived monocytes infected with *C. albicans* released ten times more hEVs than uninfected cells in a short period of time, starting after 10 min after infection and increasing over the next 20–40 min. The proteomic content of the hEVs was reported to be altered and the presence of the vesicular markers CD9 and HSP90 was detected (Halder et al., 2020). Additionally, the level of cytokines in the hEVs was determined and TGF-β1 presence was reported, together with the low levels or the lack of production of IL-1β, IL-6, and IL-10. Incubation of blood-derived monocytes differentiated into macrophages with hEVs derived from infected blood-derived monocytes resulted in the reduction of IL-6 level (Halder et al., 2020). Probably, these hEVs could play a crucial role in creating a favorable environment for *C. albicans* as commensal microorganism (Brakhage et al., 2021). Further research was focused on the miRNA content of the hEVs released by infected monocytes and identified two miRNAs ‒ has-miR-21-5p and has-miR-24-3p ‒ which were particularly involved in the mechanism of cross-species communication exploited by *C. albicans* to promote fungal growth and survival (Halder et al., 2022). The latter vesicle-enclosed molecule required the binding of the fungal cell wall component β-glucan to complement receptor 3 (CR3) and fungal cell wall mannan to Toll-like receptor 4 (TLR4) (Halder et al., 2022). The varied consequences of hEVs released by different types of host cells infected with *C. albicans* on the further responses of other types of host cells are summarized in Table 2.

**Table 2:** Effects of hEVs released by *Candida*-infected host cells on the subsequent response of other types of host cells.

***Candida*-infected host cells releasing hEVs**	**Host cells responding to hEVs**	**Host response**	**Reference**
Skin-derived keratinocytes infected with *C. albicans*	Dendritic cells	Inhibition of receptors Siglec-7 and Siglec-9	Kobiela et al., 2022
THP-1 macrophage-like cells infected with *C. albicans* SC5314	THP-1 macrophage-like cells	Activation of ERK and p38 kinases;I ncrease in the production of TNF-α, IL-8 and IL-12;I ncreased candidacidal activity	Reales-Calderón et al., 2017
	THP-1 monocytes	Increase in the production of TNF-α, IL-8 and IL-12	
Blood-derived monocytes infected with *C. albicans* SC5314	Blood-derived monocytes differentiated into macrophages	Decrease in the level of IL-6	Halder et al., 2020

The effect of *Candida* on hEVs has also been studied in non-human hosts. The changes observed in the composition of hEVs released by the cells of mice eyeball infected with *C. albicans* showed upregulation of 37 proteins and downregulation of 5 proteins in comparison to EVs from uninfected cells of mice eyeball (Gandhi et al., 2022). Most of the upregulated proteins were involved in the processes of cell adhesion, cellular response, or cytoskeleton organization. The size of EVs from infected cells was comparable to control EVs, although the concentration was significantly increased. The Western blot analysis showed an enrichment of exosomal markers, including CD9, CD63 and CD81. Quantification of the level of cytokines present in hEVs produced by infected cells revealed the increase of IL-1β and IFN-γ in comparison to control EVs, after 24 h and 72 h, and slightly increased levels of IL-6 and TNF-α (Gandhi et al., 2022).

## 4 Effect of *Candida* EVs on fungal infection and host response

The communication between host cells and pathogens mediated by EVs is thought to be based on the contact and subsequent internalization of vesicles by acceptor cells; however, although potential uptake mechanisms have been proposed, including endocytosis, phagocytosis, macropinocytosis, and direct fusion with the cell membrane, none of these have yet been confirmed for *Candida* EVs affecting human cells (Sabatke et al., 2023). Nevertheless, recent studies have repeatedly demonstrated, that the cargo of EVs can influence and alter the state of their acceptor cells. The amount and the composition of vesicles released to the external environment depend on i) available protein regulators; ii) culture conditions, including thermal or oxidative stress, hypoxia, or pH changes; iii) presence of additional chemicals, such as drugs (Hahm et al., 2021). Therefore, one can hypothesize that fluctuations in the environmental conditions will also affect the communication process itself.

The initial stage of host-pathogen contact is associated with adhesion. It is therefore important to investigate the effect of EVs on this process. In studies presented by Zamith-Miranda et al. (2021), the epithelial monolayer of HeLa cells was treated for 1 h with fEVs produced by *C. albicans* or *C. auris* prior to challenging the host cells with fungal cells in order to verify the potential effect of fEVs on the *Candida* adhesion to the epithelium. For *C. auris* fEVs, a significant increase in the number of yeast cells adhered to epithelial cells was reported, whereas for *C. albicans* fEVs, there was no observable impact on the adhesion process (Zamith-Miranda et al., 2021).

Phagocytosis of pathogen cells is an important defense mechanism undertaken by host cells in response to infection. In studies described by Zamith-Miranda et al. (2021), the presence of *C. albicans* or *C. auris* fEVs for 1 h prior to infection had no significant effect on subsequent phagocytosis of *Candida* cells by murine macrophages RAW 264.7, but for bone marrow-derived macrophages preincubation with fEVs for 4 h prior to 24 h incubation with yeast cells was demonstrated to have an effect on the ability of these cells to kill *C. albicans*. For *C. auris*, fEVs were involved in the increase of fungal proliferation within host macrophages, indicating the difference in the induced effect depending on the *Candida* species (Zamith-Miranda et al., 2021).

An additional protective response of human cells to fEVs released by *Candida* is the production of cytokines. Several studies have focused on the analysis of cytokine levels following stimulation of human cells with candidal fEVs. Research performed on THP-1 cells and fEVs from *C. albicans* SC5314 showed a lack of TNF-α production by macrophage-like cells after contact with fEVs for 24 h and no significant differences between levels of IL-10 and IL-12 (Martínez-López et al., 2022). For bone marrow isolated dendritic cells (BMDC) stimulated with *C. albicans* or *C. auris* fEVs, the production of IL-12p70, TNF-α, and IL-10 was not detected, but the level of IL-6 was observed to increase after 24 h with a concomitant decrease in TGF-β, indicating the induction of an inflammatory response by dendritic cells. The expression of MHC-II, CD80 and CD86 after stimulation with fEVs obtained from *C. albicans* and *C. auris* confirmed the activation of dendritic cells to activate CD4^+^ *T*-cells (Zamith-Miranda et al., 2021).

Additionally, one of the possible reactions of host cells to fEVs important to consider is the cell damage and death. However, as mentioned above, in the studies by Wei et al. (2023) a rather low destructive potential of fEVs on epithelial cells was demonstrated when they were not accompanied by fungal cells. In the case of immune cells, macrophage-like cells differentiated from the THP-1 cell line, after contact with *Candida* EVs did not show any considerable damage, as measured by the standard laboratory tests, which may indicate that the response of human cells to fEVs is generated through another mechanism. For yeast-like cells of *C. albicans* strain SC5314, LDH release by immune cells was measured after exposure to fEVs and there were no significant changes compared to the control cells (Martínez-López et al., 2022). For fEVs released by *C. albicans* strain 10,231 grown under stress conditions, due to oxidative stress or host concentration of carbon dioxide, their cytotoxicity was measured by the XTT assay, and no cytotoxic effect on THP-1 cells was demonstrated (Kulig et al., 2023). Moreover, in the same work the internalization of fEVs ‒ both control and released under stress conditions ‒ by THP-1 cells was demonstrated with the use of vesicles labeled with tetramethylrhodamine-conjugated concanavalin A, being a lectin specific for mannose present in fungal glycoproteins (Kulig et al., 2023). The use of this type of staining was possible due to previously confirmed presence of mannoproteins on the surface of fEVs produced by different *Candida* species (Karkowska-Kuleta et al., 2020a).

Importantly, the development of fungal infection in the host environment is associated with changing host conditions in the response to pathogen virulence factors. The immunomodulatory potential of fEVs from *C. haemulonii* var. *vulera* leads to generating an oxidative response after 24 h by murine macrophages RAW 264.7 (Oliveira et al., 2023). Although there was no reduction in the viability of RAW 264.7 cells by *C. haemulonii* fEVs, the classical NOX-2 pathway was activated to elicit oxidative stress response, which resulted in increased levels of H_2_O_2_ and O_2_^•−^, but the activation of COX-2-PGE_2_ pathway was not observed (Oliveira et al., 2023). The concentration-dependence in activating the generation of oxidative burst by murine macrophages was observed, and low doses of fEVs appeared to evade host immune responses, while higher concentrations of EVs activated antimicrobial mechanisms of host cells (Oliveira et al., 2023).

Following on from the above results, the effect of altering the host environment during *Candida* infection on the responses of other host cells was investigated. For this purpose, conditions that mimic the natural host environment have been introduced for *Candida* growth, represented by oxidative stress, nutrient- or atmosphere-limited conditions, or host environment during infection, including the presence of antifungal drugs. In our previous work, the proinflammatory effect of fEVs released by yeast-like cells of *C. albicans* strain 10,231 under oxidative stress in the presence of menadione, host carbon dioxide concentration (5 %), or control conditions was demonstrated (Kulig et al., 2023). The macrophage-like cells differentiated from THP-1 cells produced IL-1β, but no significant differences were observed for three types of fEVs tested. For TNF-α, a proinflammatory cytokine, an increase in its level was shown for fEVs released under all tested conditions compared to untreated cells, although the greatest change was observed after stimulation with fEVs produced by fungi cultured under oxidative stress condition. As an indication of the rather proinflammatory properties of tested fEVs, generated under host-derived conditions, the production of IL-10 was also verified (Kulig et al., 2023), and for all three types of fEVs the level of IL-10 was lower than in untreated cells. Furthermore, macrophage-like cells responded by producing a greater amount of IL-8 after contact with fEVs released under stress and control conditions, which was the most pronounced for fEVs produced under oxidative stress conditions (Kulig et al., 2023), which could be explained by altered lipid composition of fEVs observed in other studies for fEVs produced by *C. albicans* strain ATCC 64,548 also in the presence of menadione (Trentin et al., 2023). Furthermore, the analysis of the potential of fEVs obtained from *C. albicans* strain SC5314 and mutant strains, unable to synthesize phosphatidylserine or decarboxylate phosphatidylethanolamine, to induce activation of NF-kB by bone marrow-derived macrophages (BMDM) or murine macrophage-like J774.14 cells showed the lack of this ability only for the fEVs derived from the mutant strain unable to synthesize phosphatidylserine (Wolf et al., 2015).

In the study by Konečná et al. (2019), the modification of culture conditions simulating another type of stress conditions, related to the limitation of the nutrient availability, resulted in the increase in the number of molecules that are virulence factors present in the fEVs from *C. albicans* strain ATCC 90,028 and the yeasts of the clinical isolate from the patient with vulvovaginal candidiasis (Konečná et al., 2019). The proteomic analysis revealed that for both tested strains the most represented group were proteins involved in the pathogenesis-related processes, both adhesion and virulence, and in the response to stress conditions. The number of proteins present in fEVs was significantly higher for vesicles obtained from clinical isolate. Among proteins related with pathogenesis and identified in fEVs from both *C. albicans* strains the most abundant were agglutinin-like proteins (Als2, Als4), secreted aspartic proteases (Sap2, Sap3, Sap9), glucan 1,3-β-glucosidases Bgl2 and Xog1, cell surface mannoprotein Mp65, yeast-form wall protein Ywp1, extracellular glucosidase Utr2, secreted β-glucosidase Sun41, lysophospholipases (Plb1, Plb3, Plb4.5, Plb5), heat shock protein Hsp90 or moonlighting proteins such as enolase Eno1 or glyceraldehyde-3-phosphate dehydrogenase Tdh3 (Konečná et al., 2019).

The applied treatment should also be part of the consideration of the conditions in the host organism that prevail during the infection. The influence of antifungal drug present during the growth of *C. auris* on the properties of released fEVs has been demonstrated for the caspofungin ‒ a lipopeptide that inhibits the synthesis of β-1,3-glucan, a key component of fungal cell wall (Amatuzzi et al., 2022). The importance of these studies is based on the fact that they provide an interesting context for the conditions prevailing during the treatment of fungal infections. The vesicles obtained from three tested fungal strains grown in the presence of caspofungin, were larger in diameter and had a higher concentration compared to EVs derived from control conditions. The identification of the mRNA showed that the majority of identified sequences were present in fEVs derived from all three *C. auris* strains. The proteomic analysis showed changes in fEVs cargo, with enrichment of proteins involved in processes of cytoplasmic translation, biofilm formation, β-glucan biosynthesis, or cellular response to drugs (Munhoz da Rocha et al., 2021; Amatuzzi et al., 2022).

The immunomodulatory potential of vesicles has also been demonstrated for fEVs released by non*-albicans Candida* species, including *C. glabrata* (*N. glabratus*)*, C. parapsilosis* and *C. tropicalis* (Kulig et al., 2022)*.* The release of proinflammatory cytokine TNF-α was increased after stimulation of THP-1 macrophage-like cells for 24 h with fEVs produced by biofilm structures formed by *N. glabratus, C. parapsilosis* and *C. tropicalis.* The greatest impact had vesicles derived from *N. glabratus*, and for *C. parapsilosis* and *C. tropicalis* fEVs the effect was weaker and comparable with each other. A similar trend was observed for the production of IL-8, the highest level was observed after incubation with *N. glabratus* fEVs and slightly lower for *C. parapsilosis* fEVs, while the production of IL-8 after stimulation with fEVs from *C. tropicalis* had the lowest level. The confirmation of rather proinflammatory effect of non-*albicans* fEVs was the observed lower level of anti-inflammatory cytokine IL-10 after treatment with fEVs in comparison to untreated macrophage-like cells, especially for *C. tropicalis* fEVs (Kulig et al., 2022). The variation in host cell response may be due not only to the type of responding cells, but also to distinct species of *Candida* fungi, various morphological forms, and varying culture conditions.

For a non-human host, the study performed on bone marrow-derived murine macrophages, dendritic cells and RAW 264.7 cells showed the involvement of fEVs from clinically isolated *C. albicans* strain 11 in the increase of the production of nitric oxide and cytokines IL-12p40, TGF-β, IL-10 and TNF-α (Vargas et al., 2015). Additionally, the expression of CD86 and MHC-II receptors was up-regulated for macrophages and especially for dendritic cells. Furthermore, using the *G. mellonella* model, the impact of pretreatment with fEVs on the increase in the survival rate after subsequent injection of *C. albicans* cells was shown (Vargas et al., 2015). Further studies showed the protective effect of fEVs immunization against murine candidiasis with increased levels of IL-12p70, TNF-α, IFN-γ, TGF-β, IL-4, and IL-10 (Vargas et al., 2020).

The complexity of the effects of fEVs on host cells during infection and the responses related to hEVs production by different host cell types on fungal infection are summarized in Table 3.

**Table 3:** Effects of fEVs on the host response and hEVs on the *Candida* infection.

**Source of fEVs**	**Host response**	**Type of host cells**	**Reference**
*C. albicans* SC5314 (hyphal cells)	No cytotoxic effect of fEVs alone; damage of host cells after further contact with fungal cells	RAW264.7 macrophages, HOK keratinocytes, TR146 and HGEC epithelial cells	Wei et al., 2023
*C. auris* clinically isolated strains MMC1 and MMC2	Enhancement of fungal cell adhesion	HeLa epithelial cells	Zamith-Miranda et al., 2021
	Lack of effect on the phagocytosis of fungal cells	RAW 264.7 macrophages	
	Increase in the proliferation of fungal cells	Bone marrow-derived macrophages	
	Increase in the level of IL-6; decrease of TGF-β; lack of production of IL-12p70, TNF-α and IL-10	Bone marrow-derived dendritic cells	
	Up-regulated expression of CD80, CD86 and MHC-II receptors		
*C. albicans* ATCC 90,028	No significant influence on the adhesion of fungal cells	HeLa epithelial cells	Zamith-Miranda et al., 2021
	Lack of effect on the phagocytosis of fungal cells	RAW 264.7 macrophages	
	Activation to kill fungal cells	Bone marrow-derived macrophages	
	Increase in the level of IL-6; decrease of TGF-β; lack of production of IL-12p70, TNF-α and IL-10	Bone marrow-derived dendritic cells	
	Up-regulated expression of CD80, CD86 and MHC-II receptors		
*C. albicans* SC5314	No cytotoxic effect of fEVs	THP-1 macrophage-like cells	Martínez-López et al., 2022
	Lack of production of TNF- α;N o changes in the levels of IL-10 and IL-12		
*C. haemulonii* var. *vulera*	No cytotoxic effect of fEVs	RAW264.7 macrophages	Oliveira et al., 2023
	Activation of the oxidative response by the classical NOX-2 pathway		
*C. albicans* 10,231 grown under control and stress conditions	No cytotoxic effect of fEVs	THP-1 macrophage-like cells	Kulig et al., 2023
	Internalization of fEVs		
	No changes in the level of IL-1β;I ncrease in the level of TNF-α and IL-8; decrease in the production of IL-10		
*N. glabratus* (*C. glabrata* CBS 138)*, C. parapsilosis* CDC 317 and *C. tropicalis* T1B biofilm-forming cells	Increased production of TNF-α, IL-8;R educed level of IL-10 production	THP-1 macrophage-like cells	Kulig et al., 2022
*C. albicans* clinically isolated strain 11	Increased level of nitric oxide and cytokines (IL-12p40, TGF-β, IL-10, TNF-α)	Bone marrow-derived murine macrophages, dendritic cells and RAW 264.7 cells	Vargas et al., 2015
	Up-regulated expression of CD86 and MHC-II receptors	Dendritic cells and macrophages	
	Increased survival rate during fungal infection after administration of fEVs	*G. mellonella*	
*C. albicans* clinically isolated strain 11	Increased production of IL-12p70, TNF-α, IFN-γ, TGF-β, IL-4 and IL-10 after immunization with fEVs	Mice	Vargas et al., 2020

**Source of hEVs**	**Effect on *Candida* infection**		**Reference**

Leuk-1 human oral mucosal epithelial cells	Inhibition of the growth of fungal cells and hyphae formation;L imitation in the development of the oral infection in mice		Zhao et al., 2022
Blood-derived monocytes infected with *C. albicans*	Promotion of the growth and survival of fungal cells		Halder et al., 2022

## 5 Potential applications of hEVs and fEVs in bionanotechnology and nanomedicine

The continuous development of bionanotechnology and nanomedicine, together with the intensification of research on EVs, has greatly contributed to the consideration of their potential as disease biomarkers, vaccine components, or the drug delivery vehicles, with a wide range of applications in the prevention, diagnosis, prognosis, and treatment of diverse medical conditions (Ullah et al., 2023; Liu and Hu, 2023; Liu et al., 2024). EVs are currently considered as promising biomarkers for different disorders in the human organism, including infectious diseases, as the specific molecules they carry are not only protected from environmental destructive factors, but also transported long distances from the initial site of infection or tissue dysfunction, reaching body fluids such as blood, urine and saliva, where they can be collected from patients using a relatively non-invasive methods (Raeven et al., 2018; Cifferi et al., 2021). Therefore, one could consider that individual molecules present in fEVs released by *C. albicans*, such as the claudin-like Sur7 family proteins Sur7 and Evp1 (Dawson et al., 2020), as well as the 20S proteasome complex identified specifically in hyphae-derived EVs (Martínez-López et al., 2022), could be used as indicators of fungal infections as specific fEVs components identified in body fluids. In addition, several proteins detected in EVs produced by infected human cells, including aquaporin-5 (AQP5), have also been proposed as potential biomarkers, the detection of which was presumably related to the development of fungal endophthalmitis (Gandhi et al., 2022; Gandhi and Jospeh, 2023). Despite the promising potential of fEVs or infection-associated hEVs as clinical markers, this field requires further comprehensive and rigorous investigation to establish the validity and reliability of EVs for their possible integration into routine clinical practice.

Good biocompatibility, the possibility of using targeted fusion with the recipient cells and the ability to transport and protect the cargo over long distances make EVs of various origin considered as effective drug carriers (Kim et al., 2024). In the case of fungal vesicles, one of the proposed novel therapeutic approaches is the application of EVs produced by the probiotic strain *Saccharomyces boulardii* CNCM I-745 as effective transporters of anticancer drug doxorubicin. These EVs loaded with the therapeutic agent through the employed methodology, exhibited the capability of internalization with human intestinal cells and transferring vesicular cargo (Mierzejewska et al., 2023). However, apart from the undeniable benefits of the solutions based on the use of EVs in drug delivery, there are still many challenges that require detailed refinement. These particularly include the selection of appropriate methods for reproducible and highly efficient isolation of EVs, optimized cargo loading, ensuring specific targeting and effective fusion with recipient cells, as well as maintaining EVs stability, critical to prevent premature release of the therapeutic agents and to secure the functionality of EVs upon reaching the target site (Kim et al., 2024).

The potential of fEVs to modulate the function of the host immune system also leads to the consideration these structures as vaccine components that, when administered to individuals at risk of infection, could protect them against the development of serious fungal diseases (Nenciarini and Cavalieri, 2023). It has already been demonstrated for fEVs produced by pathogenic fungus *Cryptococcus neoformans* that they were effective in preventing cryptococcal infections when used as vaccines in a mouse model of fungal infection (Rizzo et al., 2021). Promising results have also been obtained for fEVs released by *C. albicans* yeast cells, as immunization of mice with vesicles induced full protection against disseminated candidiasis (Vargas et al., 2020). In addition, the stability of fEVs was proved after their storage at 4 °C, -20 °C, and  -80 °C, strengthening their potential to be used as vaccine components, and the pre-treatment with fEVs significantly reduced the mortality of *G. mellonella* larvae injected with *C. albicans* (Vargas et al., 2015; 2020). Since the development of a new vaccine is a complex process that involves extensive research and rigorous testing, further detailed studies elucidating mechanisms of fEVs biogenesis, cargo sorting, their composition and interactions with immune system, should address the challenges and explore the opportunities in the development of anti-*Candida* vaccines.

## 6 Conclusions and further directions

The area of research concerning the effect of host EVs on fungal pathogens, the release of EVs by yeasts, and their mutual influence in the next stages of interactions requires further comprehensive investigation. Although some interesting reports on this topic have been published in recent years and are discussed herein, still the amount of experimental data currently available is significantly limited. Understanding the interactions between hEVs and *Candida* cells could provide valuable insights into potential treatment strategies for infections caused by these fungi. Furthermore, the analysis of the reciprocal effects of released fEVs could not only update accessible potential therapies for yeast infections, but also shed light on the mechanisms of pathogenesis employed by *Candida* fungi.

There are still numerous unresolved questions in the rapidly evolving field of extracellular vesicles, starting from the mechanisms of their biogenesis, sorting and secretion, through the control of their stability, mechanisms of cargo release and targeted transport. Furthermore, the mechanisms of EVs uptake and receptors responsible for the contact of fEVs with host cells or hEVs with fungal cells should be identified. Another important question is whether hEVs inadvertently promote fungal virulence or whether they play a protective role. Additionally, it would be interesting to investigate the sequential pathway for triggering hEVs production by different cell types in response to fungal infection, including the influence of EVs released by epithelial cells infected with *Candida* fungi on several types of immune cells and on the process of vesicle release by these cells, as well as their composition and biological functions, or unraveling the mechanisms of mutual communication between different types of host immune system cells via their released EVs. These observations could broaden the perspective on the conditions that occur in the infectious milieu during the development of candidiasis. Investigating potential changes in the cargo of hEVs and fEVs, as well as the responses they induce in both host immune cells and fungal cells, could enhance our current understanding of host-pathogen interactions.

## Funding

This work was financially supported by the 10.13039/501100004442National Science Centre, Poland (grant number 2019/33/B/NZ6/02284 awarded to MR-K).

## CRediT authorship contribution statement

**Kamila Kulig:** Conceptualization, Writing – original draft. **Maria Rapala-Kozik:** Funding acquisition, Supervision, Writing – review & editing. **Justyna Karkowska-Kuleta:** Conceptualization, Writing – review & editing.

## Declaration of competing interest

The authors declare that they have no known competing financial interests or personal relationships that could have appeared to influence the work reported in this paper.

## Data Availability

No new data were created or analysed during this study.
